# Clinically meaningful improvements after gene therapy for aromatic L-amino acid decarboxylase deficiency (AADCd) in the Peabody Developmental Motor Scale, Second Edition (PDMS-2) and correlation with Bayley-III scores and motor milestones

**DOI:** 10.1186/s13023-025-03584-9

**Published:** 2025-02-07

**Authors:** Wuh-Liang Hwu, Hui-Min Lee, John Devin Peipert, Rongrong Zhang, Christian Werner, J. Rafael Sierra, Thomas O’Connell, Jonathan J. Woolley, Marjorie Crowell, Antonia Wang, Ioannis Tomazos

**Affiliations:** 1 Department of Medical Genetics and Pediatrics, National Taiwan University Hospital, National Taiwan University College of Medicine, Taipei, Taiwan; 2 Centre for Patient Reported Outcomes Research, Department of Applied Health Sciences, University of Birmingham, Birmingham, UK; 3 PTC Therapeutics Sweden AB, Askim, Sweden; 4 PTC Therapeutics Germany GmbH, Frankfurt am Main, Germany; 5 PTC Therapeutics, 500 Warren Corporate Center Drive, Warren, NJ 07059 USA; 6 Medicus Economics, Boston, MA USA

**Keywords:** Aromatic L-amino acid decarboxylase deficiency, Motor function, Cognitive function, Gene therapy

## Abstract

**Background:**

Aromatic L-amino acid decarboxylase deficiency (AADCd) is a rare genetic disorder characterized by movement disorders, motor and autonomic dysfunction, and developmental delays. The gene therapy eladocagene exuparvovec has become available in some regions; pooled clinical trial results demonstrate continuous long-term improvement in motor development and cognitive function. We sought to characterize clinically meaningful change in motor function, as measured by Total Peabody Developmental Motor Scales-Second Edition (PDMS-2) score, and assess correlations with cognition and language domains of the Bayley-III and motor milestone (MM) achievement.

**Methods:**

Data from *N* = 30 patients from three single-arm clinical studies of eladocagene exuparvovec were analyzed. Anchor-based estimation of the meaningful score difference (MSD) of Total PDMS-2 score was conducted using mean-difference and receiver operating characteristic curve (ROC) approaches. MM achievement served as the anchor defining meaningful change.

**Results:**

An MSD of 40 points for Total PDMS-2 score was selected for analysis as it yielded specificity > 0.95 using the ROC approach, and generally aligned with the mean-difference approach. Cumulative incidence analysis reflected that 50% of patients treated with eladocagene exuparvovec may achieve the MSD of 40-point change in Total PDMS-2 score at 6 months, and 86% at 18 months. Correlations between measures were of large magnitude and improved over time (Month 6: *r* = 0.599 [*p* = 0.0032]; Month 18: *r* = 0.796 [*p* = 0.0002]; Month 60: *r* = 0.861 [*p* = 0.0007]).

**Conclusions:**

The MSD of 40 points for Total PDMS-2 score enables the interpretation of changes observed in patients with AADCd, and suggests that treatment with eladocagene exuparvovec leads to significant improvements in motor and cognitive function.

## Introduction

Aromatic L-amino acid decarboxylase deficiency (AADCd) is a rare genetic neurometabolic disorder of monoamine neurotransmitter synthesis in which a deficiency of the AADC enzyme results in the inability to synthesize dopamine and serotonin [1]. In the absence of neuronal dopamine, patients experience movement disorders, including hypokinesia, dystonia, oculogyric crisis, and significant motor dysfunction; autonomic dysfunction, behavioral problems, and developmental delays also occur [2, 3].

AADCd presents early in life and encompasses a broad phenotypic spectrum, although most patients have severe disease characterized by full dependence, and profound motor impairment resulting in failure to reach developmental milestones (e.g., absence of head control) [4]. Early mortality occurs frequently, and most individuals require lifelong care [5, 6].

Until recently, management strategies for AADCd were symptomatic in nature and did not treat the underlying cause of disease [2, 7]. In recent years, gene therapy with eladocagene exuparvovec was developed, with marketing authorization granted in the European Union (EU) and United Kingdom (UK) in 2022 on the basis of positive recommendations from the European Medicines Agency (EMA) and the United Kingdom’s Medicines and Healthcare products Regulatory Authority (MHRA) [8, 9]. In 2024, it was subsequently granted marketing authorization in Israel [10] and received approval from the United States Food and Drug Administration for the treatment of AADCd [11]. Trials of eladocagene exuparvovec among pediatric patients have demonstrated an improvement in motor development and cognitive function and that therapy is well-tolerated; long-term data are available for most patients, with a follow-up period of 10 years in some cases [7, 12–14].

Analyses of pooled trial data over an extended time period (5 years) revealed continuous improvement in motor development and cognitive function, and maintenance of these effects at 5 years [7]. Patients’ motor function, when assessed using the Peabody Developmental Motor Scales - Second Edition (PDMS-2), was significantly higher than baseline at each time period assessed (1, 2, and 5 years); post-treatment PDMS-2 scores were not dependent on dose [7]. Similarly, for the two studies (AADC-010 and AADC-011) that assessed cognitive function using the Bayley Scale of Infant and Toddler Development, Third Edition (Bayley-III), significantly higher scores were observed at each time period versus baseline [7]. These improvements in motor function and development were observed in the absence of safety concerns; adverse events were generally mild or moderate in severity and resolved quickly. As reported previously, two deaths that occurred were unlikely due to the gene therapy [7, 13].

In 2023, the Food and Drug Administration (FDA) released guidance on methods by which patient experience data can be collected and submitted for drug development and regulatory decision-making [15]. The guidance recommends methods for the collection and analysis of clinical outcome assessment (COA) data, including the determination of clinically meaningful change in endpoints via the estimation of meaningful score differences (MSD) in descriptive analyses [15]. Estimation of the MSD for motor development, as measured by the Total PDMS-2 score, may enable the interpretation of improvements in this measure observed in the eladocagene exuparvovec trials and may further the understanding of the short- and long-term benefits of therapy among children with AADCd. The objectives of this study are therefore to (1) estimate the MSD of the Total PDMS-2 score using three clinical studies that investigated eladocagene exuparvovec for the treatment of patients with AADCd; and (2) estimate correlations between the Total PDMS-2 score, the PDMS-2 gross motor domain score only, the Bayley-III score, and motor milestone achievement.

## Methods

### Data source

Data from three single-arm, open-label clinical studies that investigated eladocagene exuparvovec for the treatment of patients with AADCd were analyzed using a data cut-off of July 2022. Trials included a compassionate use study (AADC-1601), phase 1/2 trial (NCT01395641; AADC-010), and phase 2b trial (NCT02926066; AADC-011) [7]. The three trials employed similar treatment protocols, with the exception of nine patients in the phase 2b trial who received a higher dose. Details on these studies were previously described elsewhere [7, 12, 13]. All studies were conducted at the National Taiwan University Hospital. All three clinical trials were approved by the appropriate research ethics committees and have been performed in accordance with the ethical standards as described in the Declaration of Helsinki [7, 12, 13]. Written informed consent was obtained from the parents of all of the patients [12, 13].

Criteria for trial participation included diagnosis of AADCd (as previously described in study publications [7, 12, 13]), classical clinical characteristics of AADCd (oculogyric crises, hypotonia, and developmental retardation), and ≥ 2 years of age or having a head circumference big enough for surgery. Patients with significant brain structure abnormality were excluded from participation.

For the present study, data extracted from the eladocagene exuparvovec trials include pre- and post-treatment assessments of the Total PDMS-2 and gross motor domain scores (excluding Reflexes subtest scores), Bayley-III cognition and language sub-scale scores, and motor milestone achievement.

### Description of study outcomes: Total PDMS-2 score

The PDMS-2 assesses gross and fine motor skills; it is validated among children from birth through age five, and consists of six subtests comprising 249 items [16–19]. Subtests include Reflexes (8 items), Stationary (30 items), Locomotion (89 items), Object Manipulation (24 items), Grasping (26 items), and Visual-Motor Integration (72 items). For children greater than 12 months of age, the Reflexes subtest is not administered, and for children less than 12 months of age, the Object Manipulation subtest is not administered. Items are scored on a 0–2 scale and summed within each subtest, which are subsequently added together to yield a total score; higher scores are indicative of better motor development. In the eladocagene exuparvovec trials, the Reflexes subtest was not assessed (8 items,16 total possible points), resulting in a Total PDMS-2 score range of 0-482 [7, 12–14].

Within the trials, the PDMS-2 was administered every 3 months in the first year after gene therapy, and every 6 months to 1 year thereafter [7]. At baseline, the mean Total PDMS-2 score across trials was 12.7 (standard deviation [SD] = 10.1, *n* = 30). Scores increased to 85.6 (SD 44.0, *n* = 25) at one year, 117.9 (SD 52.9, *n* = 23) at two years, and 126.6 (SD 61.4, *n* = 16) at five years.

For the present study, the Total PDMS-2 score and the gross motor domain score were obtained from the trials and re-analyzed for the purpose of the present study; the latter score being defined as a composite of subtest results that measure large muscle systems, including Stationary, Locomotion, and Object Manipulation [16–19].

### Description of study outcomes: motor milestone achievement

Motor milestone achievement was assessed in study AADC-1601 and served as the primary efficacy endpoint within the AADC-010 and AADC-011 trials. Milestones were based on the following four components of the PDMS-2: full head control (Stationary item 10), sitting unassisted (Stationary item 14), standing with support (Locomotion item 28), and walking with assistance (Locomotion item 34) [20, 21]. Achievement was recorded as mastery of the milestone, as indicated by a PDMS-2 item score of 2 points, and as emerging or partial mastery, as indicated by a score of 1 point. At five years, 81% of patients had achieved emergent or mastery of full head control, 75% sitting unassisted, 38% standing with support, and 13% walking with support [20].

### Description of study outcomes: Bayley-III cognitive and language domains

The Bayley-III assesses the developmental functioning of infants, toddlers, and young children aged 1–42 months [22, 23]. Domains assessed by the Bayley-III include cognitive, language (receptive and expressive), and motor (gross and fine). Each item is scored as credit (passed) or no credit (not passed) until five consecutive scores of no credit occur. Credited scores are summed to produce total raw scores for each scale, where higher scores are indicative of better developmental functioning. The cognitive and language domains of the Bayley-III were administered in the AADC-010 and the AADC-011 trials every 3 months in the first year after gene therapy, and every 6 months to 1 year thereafter [7]. The mean Bayley-III cognitive score was 12.4 (SD 4.1, *n* = 22) at baseline, 23.7 (SD 6.7, *n* = 19) at one year, 27.4 (SD 7.1, *n* = 18) at two years, and 31.2 (SD 10.2, *n* = 11) at five years, while the mean Bayley-III language score was 18.1 (SD 3.5, *n* = 22) at baseline, 24.7 (SD 2.8, *n* = 19) at one year, 26.8 (SD 4.7, *n* = 18) at two years, and 31.0 (SD 9.6, *n* = 11) at five years [7].

### Statistical analyses

#### Anchor-based methods for estimation of the MSD of the Total PDMS-2 score

Per FDA guidance, anchor-based methods can be used in the estimation of the MSD for identifying patients who may have experienced meaningful change in certain outcomes [15]. An anchor is defined as an “external variable, not derived from the COA whose scores require interpretation, for which meaningful differences are directly interpretable or already known” [15]. Meaningful differences on the variable that serves as anchor can subsequently be mapped onto differences in scores on the COA. In the present study analyzing data captured from three trials, motor milestone change served as the anchor within the analysis used to determine the MSD for the Total PDMS-2 score. These motor milestones, which comprised the primary efficacy endpoint in AADC-010 and AADC-011, were selected as the anchor based on the recognition that their achievement is considered meaningful to regulatory bodies [20]. Moreover, motor milestones are not derived from the Total PDMS-2, although they relate to specific items measured by the PDMS-2.

Total PDMS-2 and gross motor domain scores and achievement of motor milestones were assessed pre-treatment and in six-month intervals post-treatment across the three eladocagene exuparvovec trials informing the present analysis. Anchor-based estimation of the MSD of Total PDMS-2 score was conducted using mean-difference and receiver operating characteristic (ROC) curve approaches [24]. Both mastery of these motor milestones, as well as their emergence (PDMS-2 item score of 1, reflecting emerging or partial mastery), were considered in the analyses.

#### Estimation of the MSD for the Total PDMS-2 score: mean-difference and ROC approaches

For the mean-difference approach, the mean Total PDMS-2 score was calculated for each level of motor milestone (no motor function, full-head control, sitting unassisted, standing with support, and walking with assistance). To estimate the MSD, the differences between the mean Total PDMS-2 scores of adjacent motor milestones were calculated. Adjacent motor milestones included full-head control vs. none, sitting unassisted vs. full-head control, standing with support vs. sitting unassisted, and walking with assistance vs. standing with support. For the ROC approach, a logistic model was estimated to predict motor milestone improvement between visits, categorized as binary, as a function of Total PDMS-2 score change. Across the range of Total PDMS-2 score changes observed between visits, the logistic model was used to predict the probability of a motor milestone improvement for a given Total PDMS-2 score change. The predicted probability was used as a threshold for classification, allowing calculation of sensitivity (true positive rate) and specificity (1 – false positive rate) of different Total PDMS-2 score cutoffs (i.e., MSD estimates). Youden’s index (the sum of sensitivity and specificity – 1) was assessed across the range of Total PDMS-2 changes, indicating MSD estimates with the best balance of sensitivity and specificity.

#### Calculation of correlation coefficients

The following correlations and respective p-values were calculated as part of this analysis of data from the three clinical trials: Change from baseline (CFB) in Total PDMS-2 vs. CFB in Bayley-III score comprising the cognition and language domains, CFB in Total PDMS-2 score vs. CFB in Bayley-III cognition domain score, CFB in Total PDMS-2 score vs. CFB in Bayley-III language domain scores, CFB in Total PDMS-2 score vs. motor milestones achieved by age group (≤ 4 years; >4 years), and CFB in PDMS-2 gross motor domain vs. motor milestones achieved by age group (≤ 4 years; >4 years).

## Results

Data from *N* = 30 patients from the three single-arm clinical trials of eladocagene exuparvovec for the treatment of AADCd were analyzed. The study sample included *n* = 8 patients from AADC-1601, *n* = 10 from AADC-010, and *n* = 12 from AADC-011. Mean (SD) age at initiation of eladocagene exuparvovec was 45.7 (26.2) months; 53.3% were male. Follow-up for motor milestone assessments ranged from 6 to 120 months (mean: 56.6 months; median: 60 months). Further details on these study patients were previously described elsewhere [7, 12, 13].

### MSD estimation: mean-difference and ROC approaches

**Fig. 1:**
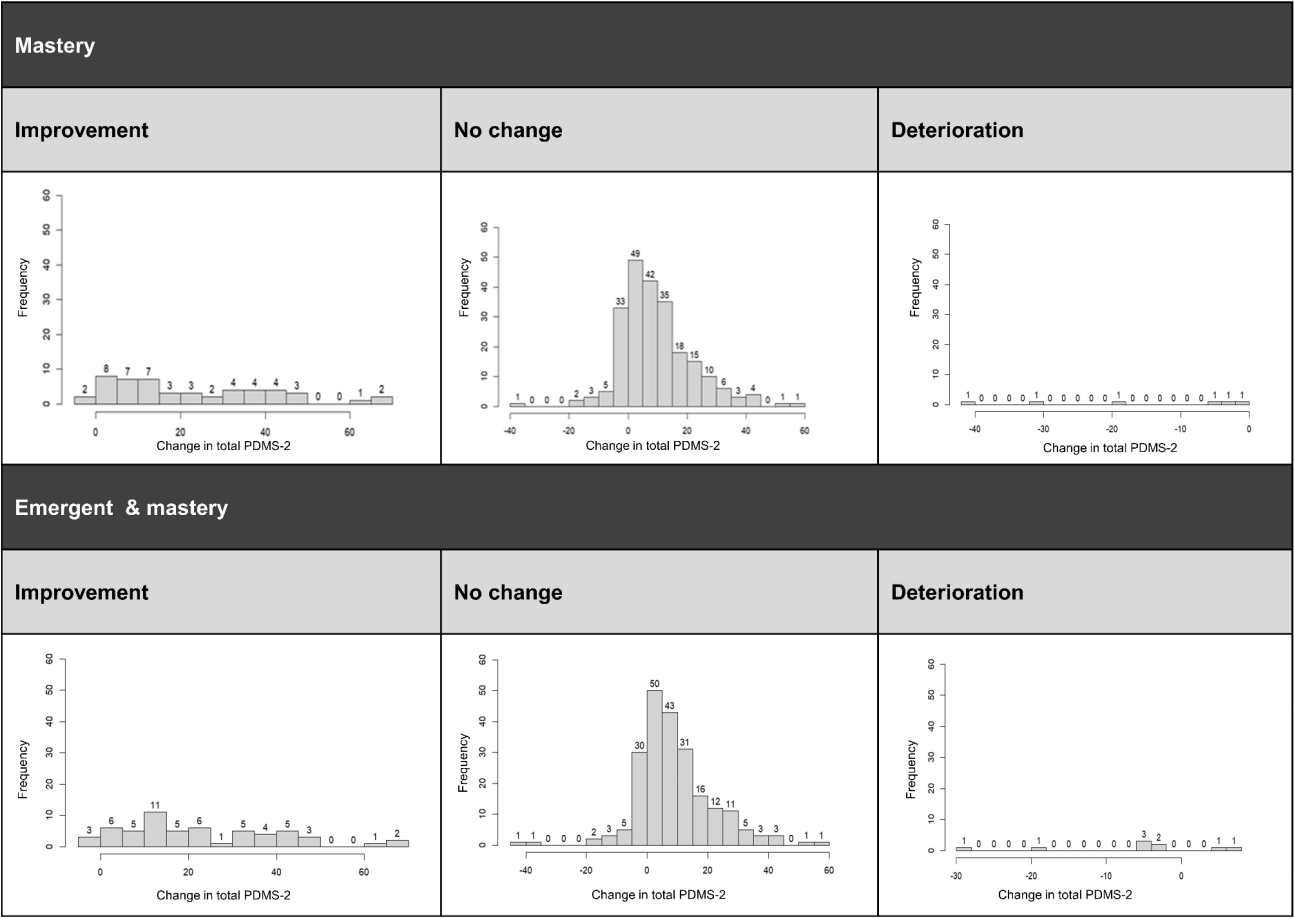
Distribution of Total PDMS-2 score change, by motor milestone change

Total PDMS-2 score and motor milestone were captured at a total of 314 visits, yielding 284 observations of change post-baseline that informed the present analysis of clinical trial data. When defining motor milestone achievement as mastery (PDMS-2 item score of 2), motor milestones were observed to improve at 50 visits, to be unchanged at 228 visits, and to have deteriorated at 6 visits. When defining motor milestone achievement as emergent or mastery (PDMS-2 item score of 1 or 2), motor milestones were observed to improve at 57 visits, to be unchanged at 218 visits, and to have deteriorated at 9 visits. Figure 1 depicts the distribution of Total PDMS-2 score change, by motor milestone change. Loss of achievement of a motor milestone, as determined based on emergence or mastery, was observed for 5 of the 30 patients through the July 2022 data cut-off. For 2 of the 5 patients, loss of a milestone reflected loss of emergence of a milestone (as reflected in Fig. 1 by the greater number of observations of deterioration for emergence and mastery vs. mastery alone). When achievement of a motor milestone was lost, it was subsequently regained at later assessments in certain cases.

**Fig. 2:**
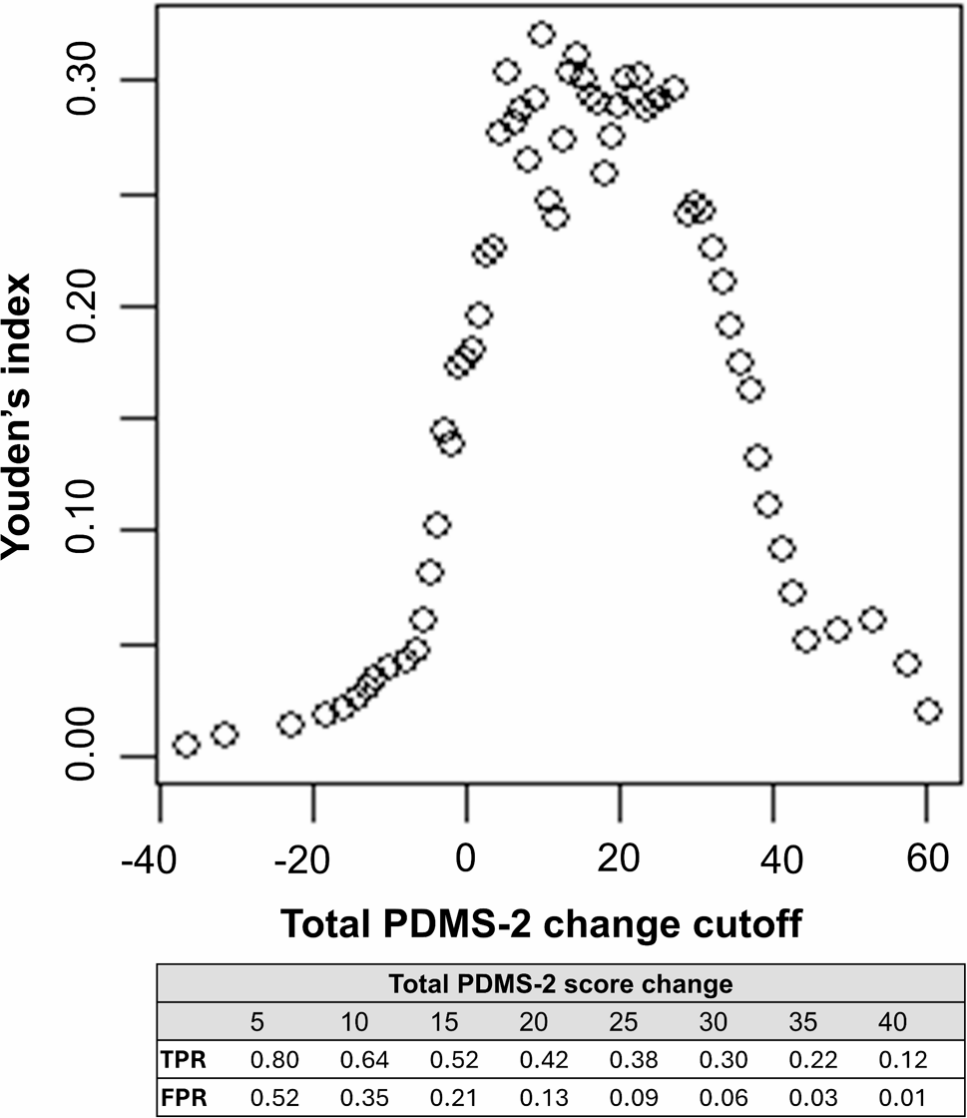
Results from the MSD analyses: ROC approach (Mastery). Abbreviations: FPR, False positive rate (1 – specificity); ROC, Receiver operating characteristic; TPR, True positive rate (sensitivity). Note: Bolded values indicate where specificity ≥ 0.95

**Fig. 3:**
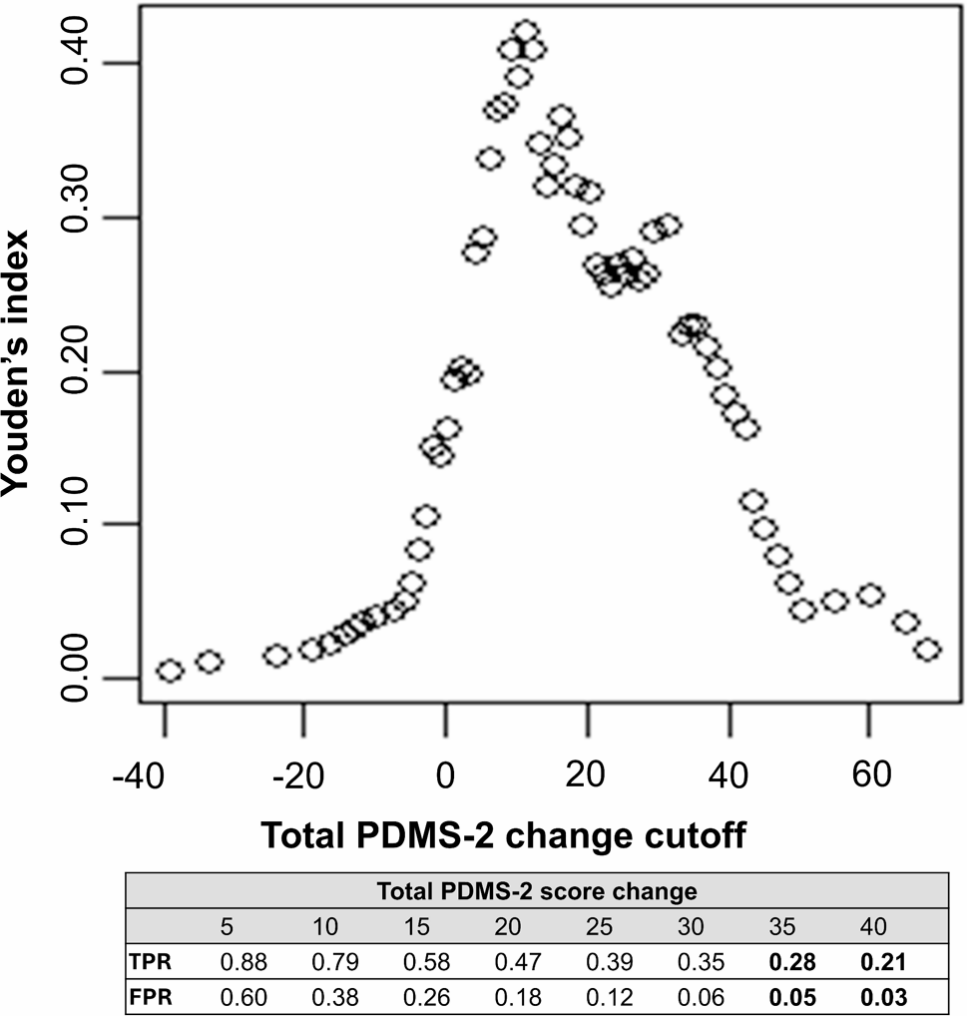
Results from the MSD analyses: ROC approach (Emergent and mastery). Abbreviations: FPR, False positive rate (1 – specificity); ROC, Receiver operating characteristic; TPR, True positive rate (sensitivity). Note: Bolded values indicate where specificity ≥ 0.95

When considering emergence or mastery of motor milestones, the MSD was estimated to be 45.6 using the mean-difference approach. When only mastery of each motor milestone was considered in the analysis, the MSD was estimated to be 45.0. Table 1 contains the results of the mean-difference MSD calculations. Using the ROC approach, the estimated MSD for the Total PDMS-2 was 30-40-points when specificity was maximized (minimizing false signals of improvement). For motor milestone improvement defined either as emergent or mastery, or mastery only, an MSD of 35–40 points yielded specificity for prediction of motor milestone improvement of ≥ 0.95. Figures 2 and 3 depict sensitivity and specificity associated with different MSD estimates in the ROC approach.

**Table 1:** Results from the MSD analyses: Mean-difference approach

	MeanTotal PDMS-2 score	Difference^1^	*N* ^2^
Motor milestone: Mastery			
None	45.0		165
Head control	91.4	46.4	38
Sitting unassisted	127.0	35.6	62
Standing with support	173.0	46.0	32
Walking with assistance	225.0	52.0	17
Mean difference: 45.0			
Motor milestone: Emergent and Mastery			
None	42.4		149
Head control	83.7	41.3	41
Sitting unassisted	117.0	33.3	69
Standing with support	170.0	53.0	38
Walking with assistance	225.0	55.0	17
Mean difference: 45.6			

^1^The difference between means of adjacent Motor milestones (full-head control vs. none, sitting unassisted vs. full-head control, standing with support vs. sitting unassisted, and walking with assistance vs. standing with support)

^2^N = number of observations

**Fig. 4:**
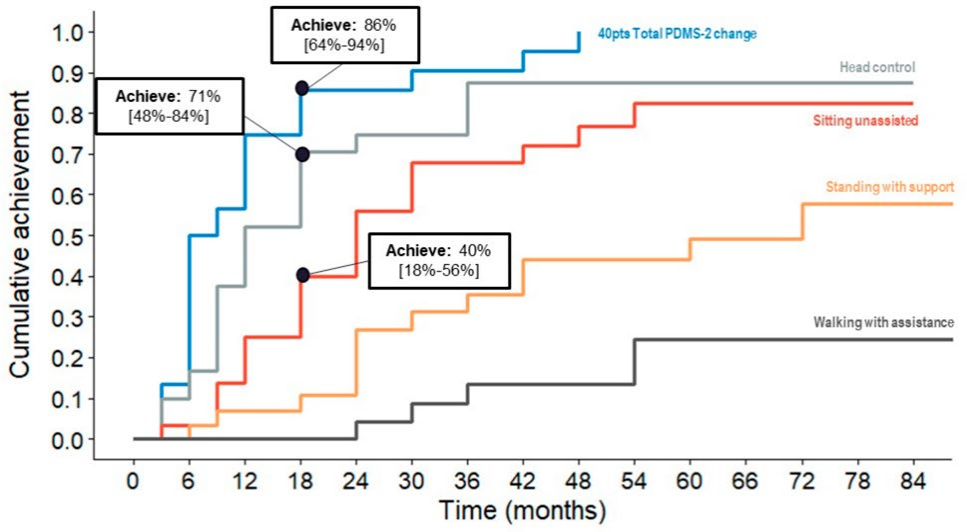
Cumulative incidence of patients achieving 40-point Total PDMS-2 score compared to proportion achieving motor milestones over time, following eladocagene exuparvovec treatment. Abbreviations: pt, points

Following MSD estimation using the mean-difference and ROC approaches, a conservative MSD of 40 points for Total PDMS-2 score was selected for analysis, as it yielded specificity > 0.95 (false positive rate < 5%) using the ROC approach and generally aligned with the estimate from the mean-difference approach (~ 45 points). In subsequent cumulative incidence analysis of achievement of the MSD of 40-point change in Total PDMS-2 score (Table 2), it was estimated that 50% of patients treated with eladocagene exuparvovec had achieved the MSD at 6 months, and 86% at 18 months. At the 18-month timepoint, 71% of patients had achieved head control and 40% were sitting unassisted. Figure 4 depicts the cumulative incidence analysis of patients achieving the 40-point change in Total PDMS-2 score, compared to motor milestones over time.

**Table 2:** Cumulative incidence and 95% CI of patients achieving the MSD of the total PDMS-2 score and each motor milestone

	Month 0	Month 6	Month 12	Month 18	Month 24	Month 30	Month 36	Month 42	Month 48	Month 54	Month 60	Month 66	Month 72	Month 78	Month 84
40pts PDMS-2 change	0 (0, 0)	50 (28, 65)	75 (53, 87)	86 (65, 94)	86 (65, 94)	90 (68, 97)	90 (68, 97)	95 (70, 99)	100 (70, 99)	100 (70, 99)	100 (70, 99)	100 (70, 99)	100 (70, 99)	100 (70, 99)	100 (70, 99)
Head control	0 (0, 0)	17 (2.2, 29)	52 (30, 67)	71 (48, 83)	75 (52, 87)	75 (52, 87)	87 (65, 96)	87 (65, 96)	87 (65, 96)	87 (65, 96)	87 (65, 96)	87 (65, 96)	87 (65, 96)	87 (65, 96)	87 (65, 96)
Sitting unassisted	0 (0, 0)	3.3 (0, 9.5)	25 (7.0, 39)	40 (18, 56)	56 (32, 71)	68 (44, 82)	68 (44, 82)	72 (48, 85)	77 (52, 89)	82 (56, 93)	82 (56, 93)	82 (56, 93)	82 (56, 93)	82 (56, 93)	82 (56, 93)
Standing with support	0 (0, 0)	3.3 (0, 9.5)	6.8 (0, 15)	11 (0, 21)	27 (7.5, 42)	31 (10, 47)	36 (14, 52)	44 (20, 61)	44 (20, 61)	44 (20, 61)	49 (24, 66)	49 (24, 66)	58 (28, 75)	58 (28, 75)	58 (28, 75)
Walking with assistance	0 (0, 0)	0 (0, 0)	0 (0, 0)	0 (0, 0)	4.2 (0, 12)	8.7 (0, 20)	14 (0, 27)	14 (0, 27)	14 (0, 27)	24 (3.0, 41)	24 (3.0, 41)	24 (3.0, 41)	24 (3.0, 41)	24 (3.0, 41)	24 (3.0, 41)

**Table 3:** Correlation coefficients between CFB Total PDMS-2 and Bayley-III (cognition and language domains) scores over time

Timepoint	*n*	Both cognition & language domains		Cognition subtest only		Receptive communication subtest only		Expressive communication subtest only	
		Correlation coefficient	*p*-value	Correlation coefficient	*p*-value	Correlation coefficient	*p*-value	Correlation coefficient	*p*-value
Month 3	22	0.251	0.2606	0.372	0.0878	0.135	0.5485	-0.277	0.2125
Month 6	22	0.599	**0.0032**	0.637	**0.0014**	0.210	0.3472	-0.132	0.5589
Month 9	20	0.735	**0.0002**	0.808	**< 0.0001**	0.223	0.3438	0.147	0.5353
Month 12	19	0.769	**0.0001**	0.858	**< 0.0001**	0.243	0.3156	0.141	0.5659
Month 18	16	0.796	**0.0002**	0.881	**< 0.0001**	0.314	0.2361	0.261	0.3288
Month 24	18	0.788	**0.0001**	0.923	**< 0.0001**	0.416	0.0862	0.309	0.2114
Month 30	16	0.847	**< 0.0001**	0.953	**< 0.0001**	0.523	**0.0376**	0.380	0.1468
Month 36	14	0.767	**0.0014**	0.945^a^	**< 0.0001**	0.730	**0.0030**	0.448	0.1084
Month 42	13	0.632	**0.0205**	0.958^b^	**< 0.0001**	0.716	**0.0059**	0.413	0.1603
Month 48	13	0.795	**0.0012**	0.926	**< 0.0001**	0.678	**0.0109**	0.299	0.3212
Month 54	10	0.834	**0.0027**	0.943	**< 0.0001**	0.757	**0.0113**	0.416	0.2315
Month 60	11	0.861	**0.0007**	0.959	**< 0.0001**	0.727	**0.0113**	0.471	0.1441
Month 72	6	0.972	**0.0012**	0.979	**0.0006**	0.836	0.0775^c^	0.593	0.2143
Month 84	4	0.997	**0.0028**	0.979	**0.0210**	0.942	0.0584	0.825	0.1747

^a^*n* = 13; ^b^*n* = 12; ^c^*n* = 5

Abbreviations: CFB, change from baseline; n, number of patients; PDMS-2, Peabody Developmental Motor Scale, Second Edition

### Correlations

Based on data from three eladocagene exuparvovec trials [7, 12, 13], correlations between CFB in Total PDMS-2 and CFB in Bayley-III scores (cognition and language domains) improved over time. Correlations were of large magnitude and statistically significant from Month 6 onwards; specifically, *r* = 0.599 (*p* = 0.0032) at Month 6, *r* = 0.796 (*p* = 0.0002) at Month 18, and *r* = 0.861 (*p* = 0.0007) at Month 60.

When examined by subtest, statistical significance of the CFB in Total PDMS-2 score vs. CFB in Bayley-III cognition subtest score was achieved by 6 months (*r* = 0.637, *p* = 0.0014). Regarding the CFB in Total PDMS-2 score vs. CFB in Bayley-III language subtests, statistical significance in the receptive communication subtest was achieved at 30 months (*r* = 0.523, *p* = 0.0376). For the expressive communication subtest, while the correlation improved over time, statistical significance was not reached. Table 3 contains the results of the correlation analyses.

Correlations between achievement in motor milestones and CFB in Total PDMS-2 score were statistically significant both for patients aged ≥ 4 (*r* = 0.934, *p* < 0.0001) and < 4 years (*r* = 0.892, *p* < 0.0001). When looking at correlations between achievement in motor milestones and the CFB in PDMS-2 gross motor domain scores only, these correlations remained statistically significant (patients aged ≥ 4 years: *r* = 0.904, *p* < 0.0001; patients aged < 4 years: *r* = 0.958, *p* < 0.0001).

## Discussion

Based on the findings from the two anchor-based approaches implemented, this study estimated an MSD of 40 points for the Total PDMS-2 score using data from three eladocagene exuparvovec clinical trials. In addition, findings showed significant correlations between CFB in Total PDMS-2 and Bayley-III cognition and receptive communication domain scores that persisted over time. Overall, the mean-difference and ROC approaches for MSD estimation generally aligned in terms of the estimates they yielded. While the mean-difference approach suggested an MSD of ~ 45 points, the ROC approach indicated that an MSD of 40 points yields specificity for prediction of motor milestone improvement of > 0.95. Accordingly, an MSD of 40 points appears to be a conservative threshold for clinically meaningful difference. Few published estimates of MSD or minimal clinically important difference (MCID) for Total PDMS-2 score are available in the literature. In such studies, MCID was generally estimated using distributional approaches (e.g., proportions of the standard deviation and/or standard error of measurement), which FDA guidance [15] recommends only for validation of estimates derived from anchor-based methods, such as those used in the present study. Previous studies also typically reported lower estimates than the MSD of 40 points identified in this study; for example, one study reported an MCID of 8.39 for children with intellectual disabilities [25]. Accordingly, the higher MSD for AADCd of 40 points estimated in this study may be conservative relative to the limited number of estimates in other disease populations, which aligns with the high specificity cutoff (i.e., minimizing false-positive predictions of meaningful change) used for selection of the MSD in our ROC analyses.

In clinical trials, AADCd patients treated with eladocagene exuparvovec experienced meaningful improvements in motor function, reflected by significant improvements in Total PDMS-2 score. By capturing a broad range of both gross and fine motor domains, the Total PDMS-2 score MSD provides greater sensitivity in measuring improvements than the five levels of motor milestones; these improvements may be notable as early as six months following treatment, before improvements are observed in motor milestones. Accordingly, the MSD of 40 points for the Total PDMS-2 score in AADCd may enable greater sensitivity for assessment of improvements observed in these studies, particularly at earlier points of assessment, while remaining reflective of patient-relevant benefit through use of the motor milestone anchor. The validity of use of the motor milestones as an anchor is underscored by the fact that this outcome was recommended as the primary endpoint for eladocagene exuparvovec trials AADC-010 and AADC-011 by the FDA (July 2017) and the EMA (December 2017) [26, 27].

Significant correlations between CFB in Total PDMS-2 and Bayley-III cognition and receptive communication domain scores suggest that in AADCd, motor function improvements measured with PDMS-2 may be associated with improvements in other domains (including non-motor domains). As cognitive function development relies on the ability of learning, while enhancing motor skills improves learning ability through increased cortical stimulation [28], improvements in cognitive function development could potentially be linked to improvements in motor skills following eladocagene exuparvovec treatment. Moreover, as the PDMS-2 and motor milestones both assess gross motor skills, a high degree of correlation is expected. Additionally, the lower correlation observed with expressive communication is likely a result of the later attainment of these skills in the course of childhood development. While the PDMS-2 has only been validated for children aged 0 to 5 years, correlations between CFB in Total PDMS-2 score and motor milestone achievement were consistent and significant for both the age ≥ 4 and < 4 years groups, which was also observed with CFB in PDMS-2 gross motor domain score and motor milestone achievement.

This study implemented methods that are aligned with FDA guidance on the estimation of MSDs and incorporated two approaches to derive an estimate for Total PDMS-2 score that maximizes specificity. As a standardized measure of motor skills that may be used to identify patients with motor deficits, Total PDMS-2 score is an important trial endpoint for evaluating novel treatments for AADCd, and potentially other ultra-rare conditions affecting motor development. As such, the estimated MSD allows for a better understanding of the clinical relevance of changes observed in Total PDMS-2 scores; these changes highlight the clinically relevant benefits of eladocagene exuparvovec for patients with AADCd. Beyond interpretation of patient-specific change in clinical practice, the MSD for Total PDMS-2 may be used to model the trajectory and progression of AADCd. Moreover, use of the MSD could be considered for informing economic models to establish the cost-effectiveness of novel therapies for AADCd.

Certain considerations should be made and limitations noted when interpreting the findings of this study. Given the rarity of AADCd, small sample sizes in the data, in particular at later time points, may impact the robustness of results for correlations between CFB in Total PDMS-2 and Bayley-III scores over time. Nevertheless, nearly 300 observations of change post-baseline were available for analysis. Next, the Reflexes subtest of the PDMS-2 was not administered in the eladocagene exuparvovec studies. Therefore, the MSD of the Total PDMS-2 score as estimated in the present study reflects a total score range from 0-482 and may only be generalizable to instances where the Reflexes subtest is excluded.

## Conclusion

The MSD of 40 points for the Total PDMS-2 score when used among patients with AADCd enables the interpretation of improvements observed in clinical studies. Findings from the current study suggest that for patients with AADCd, treatment with eladocagene exuparvovec leads to significant improvements in motor and cognitive function.

## Data Availability

The datasets generated in this study are available from the corresponding author upon reasonable request.

## Abbreviations

AADCd: Aromatic L-amino acid decarboxylase deficiency
CFB: Change from baseline
COA: Clinical outcome assessment
FDA: Food and Drug Administration
EMA: European Medicines Agency
EU: European Union
MCID: Minimal clinically important difference
MHRA: Medicines and Healthcare products Regulatory Authority
MM: Motor milestones
MSD: Meaningful score difference
PDMS-2: Peabody Developmental Motor Scale, Second Edition
ROC: Receiver operating characteristic
SD: Standard deviation
UK: United Kingdom
